# 

**Published:** 1859-01

**Authors:** 


*Subscribers and exchanges*, who have kindly forwarded to us the August number have been duly credited; but in the case of some subscribers, we have been compelled to look through the entire mail-book, to find their place of residence, as in most cases the post-mark has been absolutely illegible. Those who may send us any more copies would save us considerable trouble, if they would put down their post office, as well as the name.

*Professional Changes.*—Dr. Jas. B. McCaw, well and favorably known as one of the editors of the "Virginia Medical Journal," has been elected to the chair of Chemistry and Pharmacy in the "Virginia Medical College," Richmond, Va. We have been most favorably impressed with him as an editor, and have no doubt, but that he will make an equally able teacher.

Dr. May's position in the "National Medical College," at Washington, D. C., has been supplied by Dr. J. G. F. Holston, who, we have every reason to believe, will prove a most excellent instructor.—*Savannah Journal of Medicine.*

---

Prof. Flint has promised us a letter for insertion in the Journal, giving an account of medical matters in New Orleans. We hope to receive it in season for insertion in our next issue.

*Erie County Medical Society.*—The annual meeting of the Medical Society of the county of Erie will be held in this city, on Tuesday, January 11th, 1859, at the rooms of the Buffalo Medical Association, No. 7 South Division street, at 10½ o'clock, A. M.

JAMES M. NEWMAN, Secretary.

Buffalo, Dec. 25th, 1858.

We would call the attention of the profession of the county to the above notice of the Secretary, and especially of our country brethren, hoping thereby to secure a more full atendance of their numbers than has been the practice for several years past. It should be remembered, that membership with the County Society constitutes the great dividing line between regular and irregular practitioners, and our country practitioners, who are desirous of preserving these lines of distinction, should manifest sufficient interest in the Society to attend its meetings. At the same time, we would exhort those who desire to be recognized as within the pale of the regular profession, and have heretofore neglected to do so, to make such a declaration, by a union with the Society. We regret to say, that very many practitioners in the country, who would repel the idea of being called irregulars, have neglected to comply with the requirement of the laws, and become members of the Society. A good opportunity to remove any imputations of the kind, will be afforded at the coming annual session.

At the semi-annual meeting in June, a committee was appointed to make arrangements for an annual dinner at the present meeting; and, it is, we presume, unnecessary to say, that the city members will be most happy to receive and entertain as many of our country brethren, in regular connection with the Society, at the festivities with which it is proposed to close the labors of the day, as may find it convenient to attend.

---

☞ Prof. Flint left Buffalo in the early part of November to enter on the duties of his Chair in the New Orleans School of Medicine. He expects to return in the latter part of February next. His correspondents, in the meantime, will please direct their letters to him at New Orleans.

